# Extracellular vesicles from *Lactobacillus plantarum* alleviate DNFB-induced atopic dermatitis in mice by reconstructing the skin barrier and inhibiting inflammation

**DOI:** 10.3389/fimmu.2026.1873193

**Published:** 2026-08-11

**Authors:** Qingying Shi, Fanglu Yu, Maoyuan Zhou, Hongmin Niu, Hong Zhao, Mohan Xu, Linbang Long, Zhepeng Cao, Xinyue Yu, Yanxiang Jiang, Fufeng Liu, Fuping Lu, Zhengmei Huang, Xihong He, Huabing Zhao

**Affiliations:** 1 Key Laboratory of Industrial Fermentation Microbiology, Ministry of Education, Tianjin Key Laboratory of Industrial Microbiology, College of Biotechnology, Tianjin University of Science and Technology, Tianjin, China; 2 Tianjin Customs Animal, Plant, and Food Testing Center, Tianjin, China; 3 Onlystar Biotechnology Co., Ltd., Dezhou, China

**Keywords:** 11-trans-octadecenoic acid, atopic dermatitis, bacterial extracellular vesicles, inflammation, *Lactobacillus plantarum*, pruritus, skin barrier

## Abstract

**Introduction:**

Atopic dermatitis (AD), a chronic inflammatory skin disorder, represents a significant global health challenge. Although probiotic therapy shows potential for skin disease management, the application of live bacteria remains associated with multiple limitations. Bacterial extracellular vesicles (EVs) may provide a safer and more effective alternative.

**Methods:**

In this study, EVs derived from *Lactobacillus plantarum* were isolated and characterized. Their therapeutic potential against AD was evaluated through *in vitro* and *in vivo* experiments. HaCaT keratinocytes were used to establish an AD-like cellular model, and an AD mouse model was induced using 2,4-dinitrofluorobenzene (DNFB). The effects of *L. plantarum* EVs on skin barrier function, inflammatory responses, and scratching behaviors were evaluated. Multi-omics analysis was performed to explore the potential bioactive components within EVs underlying their therapeutic effects.

**Results:**

*In vitro*, *L. plantarum* EVs were efficiently internalized by HaCaT keratinocytes, significantly increasing ceramide levels and upregulating key barrier-related genes, including filaggrin (FLG), desmoglein-1 (DSG1), and ceramide synthase 3 (CerS3), thereby enhancing skin barrier function in the AD-like cell model. Meanwhile, EVs treatment downregulated pro-inflammatory cytokines, including thymic stromal lymphopoietin (TSLP), interleukin-25 (IL-25), interleukin-33 (IL-33), and tumor necrosis factor-α (TNF-α). In the DNFB-induced AD mouse model, EVs administration markedly alleviated clinical symptoms, reduced lesion scores and transepidermal water loss, and restored epidermal thickness to near-normal levels, indicating barrier repair in AD mice. Skin tissue analysis further confirmed reduced inflammatory cytokine expression. Additionally, EVs suppressed scratching behaviors by inhibiting the IL-31 pathway. Multi-omics analysis suggested that the therapeutic effects of *L. plantarum* EVs may be associated with lipid-based bioactive components within EVs.

**Discussion:**

These findings indicate that bacterial EVs represent a promising probiotic-inspired strategy for AD treatment and provide new insights into the development of safe and effective dermatological therapies.

## 1 Introduction

Atopic dermatitis (AD) is a chronic inflammatory skin disease characterized by recurrent eczematous lesions and intense pruritus, and has been identified as one of the most common skin disorders in industrialized nations (1). Epidemiological studies indicate that AD affects approximately 10–20% of children and 5–10% of adults worldwide, with a persistently increasing prevalence (2). The AD lesion presents as itching, erythematous plaques and papules, resulting in scratch marks and serous exudate. AD is also associated with a significantly elevated risk of various comorbidities, including sleep disorders, anxiety and depression, food allergies, and asthma, all of which collectively diminish quality of life of patients while creating significant socioeconomic burdens (3, 4). The substantial disease burden underscores the urgent need to develop effective therapeutic strategies for AD.

Conventional therapies of AD primarily rely on topical corticosteroids, calcineurin inhibitors, and systemic immunosuppressants. While effective for short-term symptom relief, long-term use is associated with adverse effects including skin atrophy, systemic immunosuppression, and potential carcinogenic risks (5, 6). Consequently, there is an urgent clinical need to develop safe, effective, and sustainable alternative strategies. Probiotic therapy, involving the supplementation of skin or gut commensals to restore microbial balance and modulate host immunity, has been extensively explored for AD prevention and adjunctive treatment (7, 8). As reported, probiotics have been demonstrated to effectively alleviate AD. For example, Gueniche et al. reported that *Lactobacillus paracasei* promoted recovery of impaired skin barrier function (9). Additionally, *Bifidobacterium longum* lysates have been shown to enhance skin reactivity and mitigate cutaneous inflammation by modulating the skin microbiome and reinforcing barrier integrity (10). However, the application of live bacteria is hampered by multiple challenges including low colonization efficiency, production of harmful metabolites, susceptibility to immune system clearance, potential infection risks, cold chain transportation requirements, and limited shelf stability (11).

Extracellular vesicles (EVs) are 20–400 nm lipid bilayer nanovesicles actively secreted by bacteria. They are enriched with proteins, lipids, nucleic acids, and metabolites, enabling cross-species communication without the presence of live bacteria (12, 13). Interestingly, EVs can perform similar physiological functions as the parent cells. However, compared to their parental live bacteria, EVs offer distinct advantages, including non-replicative nature, low immunogenicity, enhanced skin permeability, and high biosafety, positioning them as ideal “cell-free” vehicles for microbial interventions (14, 15). Recent studies have demonstrated that EVs derived from *Bifidobacterium* can trigger the expression of filaggrin and loricrin in human primary epithelial keratinocytes, thereby enhancing the skin barrier function. EVs from *Lactobacillus* have also been reported to exert multi-target therapeutic effects against skin disease through pathogen inhibition, anti-inflammatory activity, and skin microbiota modulation (16). Also, *S. epidermidis*-derived EVs significantly ameliorated AD-like lesions by upregulating antimicrobial peptide expression and inhibiting the TLR2/NF-κB signaling pathway (17, 18). Therefore, the therapy based on the EVs of probiotics holds considerable promise for treating AD skin.

*Lactobacillus plantarum*, a Generally Recognized as Safe (GRAS) strain, has demonstrated significant biological regulatory functions beneficial to human health. Studies have shown that *L. plantarum* administration markedly improved AD, potentially by promoting production of key epidermal structural components and downregulating Th2-mediated inflammation, thereby disrupting the characteristic cycle of barrier dysfunction and inflammation (19). Notably, the EVs secreted from *L. plantarum* were enriched with immunologically active molecules such as lipoteichoic acid (LTA), peptidoglycan, and exopolysaccharides (20, 21). These nanosized EVs exhibited remarkable biodistribution capabilities, evidenced by their ability to cross the blood-brain barrier and modulate neuronal activity in murine models, resulting in measurable improvements in depression-like behaviors (22). Moreover, emerging *in vitro* studies confirmed that *L. plantarum* EVs can activate macrophages via the TLR2/NF-κB axis, suppressing the production of TNF-α and IL-6, indicating significant anti-inflammatory potential (23). Nevertheless, direct evidence remains lacking regarding the efficacy of *L. plantarum* EVs in alleviating AD skin inflammation and repairing barrier function *in vivo*, particularly concerning their key bioactive components and precise mechanisms of action.

Therefore, we isolated and characterized the EVs from *L. plantarum*, detailing their physicochemical properties this study. An *in vitro* AD-like cellular model was established to validate the regulatory effects of *L. plantarum* EVs on skin barrier function and inflammatory responses. Subsequently, a 2,4-dinitrofluorobenzene (DNFB)-induced mouse AD model was employed *in vivo* to further evaluate the therapeutic efficacy. Also, through integrated multi-omics approaches, we further explored the candidate bioactive components potentially responsible for these effects and validated a representative candidate, while elucidating their potential mechanistic target. Our findings stressed the significant efficacy of *L. plantarum* EVs in AD therapy, proving a novel treatment strategy for AD individuals.

## 2 Materials and methods

### 2.1 Preparation of EVs derived from *Lactobacillus plantarum*

*Lactobacillus plantarum* used in this study was isolated and preserved from kimchi samples in our laboratory. The strain was inoculated into MRS liquid medium (Oxoid, UK) and incubated at 37 °C for 16 hours. The culture medium was centrifuged at 4 °C for 20 minutes at 6,000 × g to remove bacterial cells. The supernatant was subsequently filtered through filter membranes with pore sizes of 0.45 μm and then 0.22 μm (Millipore), followed by concentration using an ultrafiltration device set. The concentrated solution was then subjected to centrifugation at 150,000 × g for 1.5 hours using an SW 32 Ti rotor (Beckman Coulter). The resulting precipitate was resuspended in sterile phosphate buffer solution (PBS), sterilized through a 0.22 μm filter membrane and stored at -80 °C.

### 2.2 Characterization of EVs

Transmission Electron Microscopy (TEM) was used to observe the morphological characteristics of EVs. A volume of 10 μL of EVs was placed on a carbon-coated copper grid followed by air drying, and then negative staining was performed using a solution of phosphotungstic acid at a concentration of 2% for 5 minutes. After drying at room temperature, observations were made under the TEM (Hitachi, Japan). The nanoparticle tracking analysis (NTA) were conducted to measure the size distributions and particle concentration of EVs by NanoSight NS300 (Malvern, UK). The Zeta potential of EVs was determined by Zetasizer Nano ZS90 (Malvern, UK). The protein concentration of EVs was measured by Bicinchinonic acid (BCA) Protein Assay Kit (Beyotime, China) according to the instructions of the manufacturer. The method of Sodium dodecyl sulfate polyacrylamide gel electrophoresis (SDS-PAGE) analysis was consistent with that documented in the previous study (24).

### 2.3 Cell culture

The human immortalized keratinocyte cell line HaCaT (ATCC) was routinely cultured in high-glucose (4.5 g/L glucose) Dulbecco’s Modified Eagle Medium (DMEM) supplemented with 10% (v/v) fetal bovine serum (FBS, Gibco) and 1% penicillin-streptomycin. The cells were maintained in an incubator at 37 °C with 5% CO_2_. Subculturing was performed every 2 to 3 days, the cells in the logarithmic growth phase were utilized for experimentation.

### 2.4 Cell uptake assay

The EVs were labeled with green fluorescent dye DiO (Beyotime, China). Specifically, 1 μg/mL of DiO was incubated with EVs at 37 °C in the dark for 30 minutes, followed by ultracentrifugation at 150,000 × g for 1.5 hours at 4 °C to remove any unbound dye. HaCaT cells were seeded into confocal dishes at a density of 2 × 10^5^ cells per well. After allowing sufficient time for adhesion, DiO-labeled EVs were added and incubated at 37 °C for an additional 2 hours. The cells were then gently washed three times with PBS, fixed using a solution of paraformaldehyde (4%) for 15 minutes, stained with 2-(4-Amidinophenyl)-6-indolecarbamidine dihydrochloride (DAPI) for 5 minutes to visualize nuclei, and subsequently observed under a confocal laser scanning microscope (Zeiss LSM 880).

### 2.5 Cell viability detection

The cell viability was assessed using the Cell Counting Kit-8 (CCK-8) assay method. HaCaT cells were plated in a total of 96 wells at a density of approximately 5 × 10³ cells per well. Following adherence, varying concentrations of EVs ranging from 6-30 μg/mL(1.83 × 10^9^-9.14 × 10^9^ particles/mL) were introduced into the culture medium and allowed to incubate for 24 hours. Subsequently, 10 μL of CCK-8 reagent was added to each well and further incubated in the dark at 37 °C for 2 hours. The absorbance at 450 nm (OD_450_) was determined by a microplate reader.

### 2.6 Ceramide content determination

After treatment with different concentrations of EVs (6, 12, and 30 μg/mL) over 24 hours, supernatants from HaCaT cell cultures were collected for analysis. The ceramide levels within these culture supernatants were quantified utilizing the human ceramide enzyme-linked immunosorbent assay (ELISA) Kit (MyBioSource), following the manufacturer’s instructions meticulously.

### 2.7 Construction of AD-like cell model

HaCaT cells were seeded into 6-well plates. Once the confluence reached approximately 80%, the interleukin-4 (IL-4) and interleukin-13 (IL-13) were added at concentrations of 50 ng/mL each (PeproTech) to co-stimulate the cells for a duration of 24 hours, thereby establishing an inflammatory microenvironment model representative of AD.

### 2.8 Measurement of transepithelial electrical resistance

HaCaT cells were plated in a Transwell chamber with a pore size of 0.4 μm (Corning) at a density of 1 × 10^5^ cells per well and cultured for 7 days until a complete monolayer was formed. Following this period, the cell monolayers were treatment with IL-4 and IL-13 to induce inflammatory response and the EVs with various concentration was administered. Then, the transepithelial electrical resistance (TER) was measured using a Millicell-ERS voltmeter.

### 2.9 Determination of gene expression by qPCR

The expression levels of genes involved in ceramide synthesis, barrier function and inflammation response, including serine palmitoyltransferase (SPT), sphingomyelinase (SMase), ceramide synthase 3 (CerS3), and glucocerebrosidase (GCS), filaggrin (FLG), desmoglein-1 (DSG1), interleukin-25 (IL-25), interleukin-33 (IL-33), thymic stromal lymphopoietin (TSLP), tumor necrosis factor-α (TNF-α), were determined by quantitative polymerase chain reaction (qPCR). Specifically, cells treated with EVs were collected, and total RNA was extracted using the Trizol method, followed by reverse transcription to synthesize complementary DNA (cDNA). The mRNA expressions of were quantified using qPCR. The primer sequences are detailed in **Supplementary Table 1**, with Glyceraldehyde-3-phosphate dehydrogenase (GAPDH) serving as the internal reference gene. Reaction conditions included an initial denaturation at 95 °C for 3 minutes, followed by 40 cycles of denaturation at 95 °C for 10 seconds and annealing/extension at 60 °C for 30 seconds.

### 2.10 Animals and treatments

Seventy BALB/c mice (SPF grade, 6–8 weeks old, 18 ± 2 g, female) were procured from SPF (Beijing) Biotechnology Co., Ltd. All mice were housed in specific pathogen-free facilities with ad libitum access to food and water, and maintained with a cycle of 12 hours of light and 12 hours of darkness. All animal procedures were approved by the Experimental Animal Ethics Committee of Tianjin International Biomedical United Research Institute. After a 7-day acclimation period, all mice were randomly divided into 7 groups. Except for the normal control group, mice were sensitized by uniform application of 1% 2,4-dinitrofluorobenzene (DNFB) (100 μL, acetone:olive oil = 3:1, v/v) on their dorsal skin on days 0, 2, and 5. Then, the mice were challenged with 0.2% DNFB (50 μL) on days 8, 11, 13, 15, 17, and 21 to establish the AD-like mouse model. At 30 min after each challenge, 100 μL of the corresponding test substance was topically applied according to the group assignments. The normal control group and DNFB-induced model group received PBS, the positive control group was treated with 0.1% mometasone furoate cream (MFC), and the EVs-treated groups received EVs at concentrations of 6, 12, 30 and 100 μg/mL (1.83 × 10^9^, 3.66 × 10^9^, 9.14 × 10^9^ and 3.05 × 10^10^ particles/mL), respectively.

### 2.11 Clinical evaluations and biophysical measurements

The severity of dorsal skin lesions in mice was assessed on days 0, 1, 4, 7, 10, 12, 14, 16, and 20 based on four clinical parameters: erythema/hemorrhage, edema, dryness, and scaling/erosion. Each parameter was graded on a scale of 0 to 3 (0: absent; 1: mild; 2: moderate; 3: severe). On day 20 after treatment, mouse scratching behavior was assessed. Individual mice were placed in a sound-attenuated observation chamber and filmed for 10 minutes using a digital video system to quantify scratching episodes.

On day 24 after treatment, the epidermal hydration was measured using a Corneometer CM825 and assessed transepidermal water loss (TEWL) with a Tewameter TM300 under standardized conditions (24 ± 1 °C, 50-55% RH).

### 2.12 *In vivo* imaging of EVs

100 μL of free DiO and DiO-labeled EVs were topically applied to the dorsal skin of the mice to analyze the entry of EVs into the skin. The fluorescence signal of DiO in the mice was detected using the IVIS Spectrum *in vivo* imaging system (PerkinElmer) at 0 hours and 2 hours.

### 2.13 Histopathological examination

Mice were firstly anesthetized by inhalation of isoflurane at a concentration of 4% (v/v) in a sealed anesthesia chamber until loss of consciousness and pain reflexes. Then cervical dislocation was rapidly performed to confirm complete death. Following euthanasia, skin lesions from the dorsal region of the mice were excised and fixed in 4% paraformaldehyde for 24 hours. Subsequently, the tissues were embedded in paraffin and sectioned continuously at a thickness of 5 μm. Hematoxylin and eosin (H&E) staining was performed to evaluate the epidermal thickness and the extent of inflammatory cell infiltration.

### 2.14 Determination of gene expression in skin tissues by qPCR analysis

The total RNA was extracted from skin tissue samples using the Trizol method. Reverse transcription was then performed, followed by qPCR to analyze the expression levels of various target genes. The primer sequences used for amplification are provided in **Supplementary Table 2**. The genes of interest included inflammatory factors such as IL-4, TNF-α, TSLP, barrier function-related genes such as keratin 10 (KRT10), FLG, DSG1, as well as pruritus mediators such as interleukin 31 receptor A (IL-31RA) and oncostatin M receptor (OSMR).

### 2.15 Proteomics analysis

The purified EVs were resuspended in pre-cooled PBS, aliquoted into 2 mL low-adsorption centrifuge tubes, and processed in triplicate. Protein concentration was quantified using the BCA method to ensure that each sample contained at least 200 μg/mL protein. Subsequent analyses were performed by Shanghai Zhongke Xinshengsheng Biotechnology Co., Ltd., according to the following standardized procedures: (1) protein lysis followed by reduction, alkylation, and tryptic digestion; (2) separation and mass spectrometry detection using LC-MS/MS (Q-Exactive HF-X, Thermo); (3) database searching against *Lactobacillus plantarum* (UniProt) using MaxQuant (v1.6.17) with an FDR threshold of 1%; (4) differentially expressed proteins identification using Perseus, and functional annotations and pathway analysis via Gene Ontology (GO) and Kyoto Encyclopedia of Genes and Genomes (KEGG).

### 2.16 Metabolomics analysis

With a protein concentration ≥200 μg/mL, six biological replicates were prepared. The samples were submitted to Shanghai Meiji Biomedical Technology Co., Ltd. for processing, which included the following steps: (1) addition of four volumes of pre-cooled methanol-acetonitrile-water (2:2:1, v/v/v), followed by vortexing for 30 seconds; (2) rapid freezing in liquid nitrogen for 3 minutes and ultrasonic treatment in an ice-water bath for 5 minutes (40 kHz, 100 W); (3) centrifugation at 4 °C at 12,000 × g for 15 minutes, with the supernatant collected; (4) LC-MS/MS analysis in both positive and negative ion modes using UHPLC-Q-Exactive HF-X; (5) peak extraction, metabolite identification (mzCloud and HMDB databases), and pathway enrichment analysis were performed using Compound Discoverer 3.3.

### 2.17 Molecular docking

The binding efficacy between the active compound in EVs and the core target protein was analyzed using molecular docking techniques. The amino acid sequence of peroxisome proliferator-activated receptor γ (PPARγ) was first retrieved from the UniProt database (UniProt ID: P37231), and the structures of PPARγ (PDB ID: 7AWC) obtained from the Research Collaboratory for Structural Bioinformatics Protein Data Bank (RCSB PDB) database (https://www.rcsb.org/). Initial structure prediction was attempted using AlphaFold2 (AF2), but due to unsatisfactory model confidence scores, we subsequently employed homology modeling through Swiss-Model using 7AWC as the template, which yielded a reliable structure with excellent similarity to the reference (**Supplementary Figure 1**). The ligand structure of 11-trans-octadecenoic acid (VA) (CAS ID: 693-72-1) was acquired from PubChem database (https://pubchem.ncbi.nlm.nih.gov/). Prior to docking simulations, both the receptor (PPARγ) and ligand (VA) structures were processed and converted to pdbqt format using AutoDockTools (ADT). Molecular docking was performed using AutoDock Tools with the docking grid box centered on residue Y501, encompassing the binding pocket with dimensions of 17.17 Å (X) × 32.04 Å (Y) × 11.44 Å (Z). Typically, a docking energy threshold below -5 kcal/mol was considered indicative of significant binding affinity between the target protein and ligand compound (25). PyMOL software was employed to visualize the molecular interaction between the protein and ligand.

### 2.18 Statistical processing

All data are presented as mean ± standard deviation (SD). One-way ANOVA was performed using GraphPad Prism 9 (GraphPad Software, CA, USA), followed by the LSD test for multiple comparisons between groups. A p-value < 0.05 was considered statistically significant.

## 3 Results

### 3.1 Preparation and characterization of EVs derived from *Lactobacillus plantarum*

**Figure 1:**
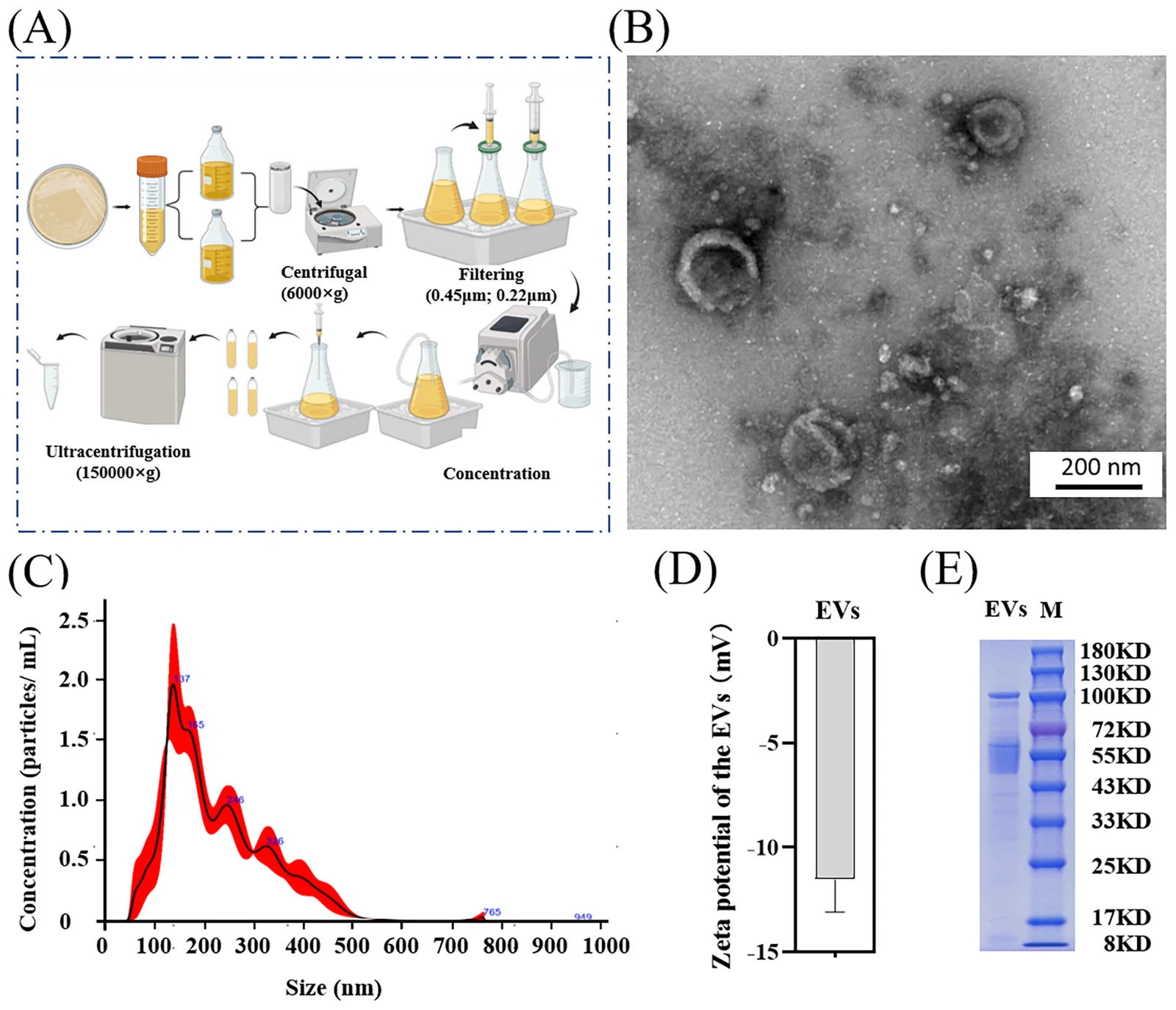
The preparation and characterization of EVs derived from *Lactobacillus plantarum*. **(A)** The specific protocols for the separation and preparation of EVs. **(B)** The transmission electron microscopy (TEM) image of EVs. **(C)** The nanoparticle tracking analysis (NTA) analysis of EVs. **(D)** The zeta potential of the EVs. **(E)** The molecular weight distribution of protein in EVs.

In this study, the EVs were isolated and prepared using ultracentrifugation after obtaining the bacterial culture (**Figure 1A**). Then, the morphological features of the EVs were observed using TEM. It’s revealed that the EVs exhibited a spherical shape and membrane structure (**Figure 1B**), consistent with the typical morphological characteristics of most reported vesicles (26, 27). The NTA analysis demonstrated that the EVs had an average diameter of 132.2 ± 5.8 nm and a concentration of 3.2 × 10^9^ particles/mL (**Figure 1C**), indicating that the isolation and purification methods used in this study efficiently yielded EVs. The mean zeta potential of the EVs was measured at -11.54 ± 1.27 mV, reflecting their surface charge stability (**Figure 1D**). The total protein content of the extracted EVs was accurately measured using the BCA protein assay, providing fundamental data for subsequent application and evaluation. Additionally, SDS-PAGE analysis revealed distinct protein bands within the EVs, with molecular weights ranging from 25 to 100 kDa (**Figure 1E**), highlighting their diverse protein composition (**Figure 1F**).

### 3.2 Cellular internalization and biocompatibility of EVs

**Figure 2:**
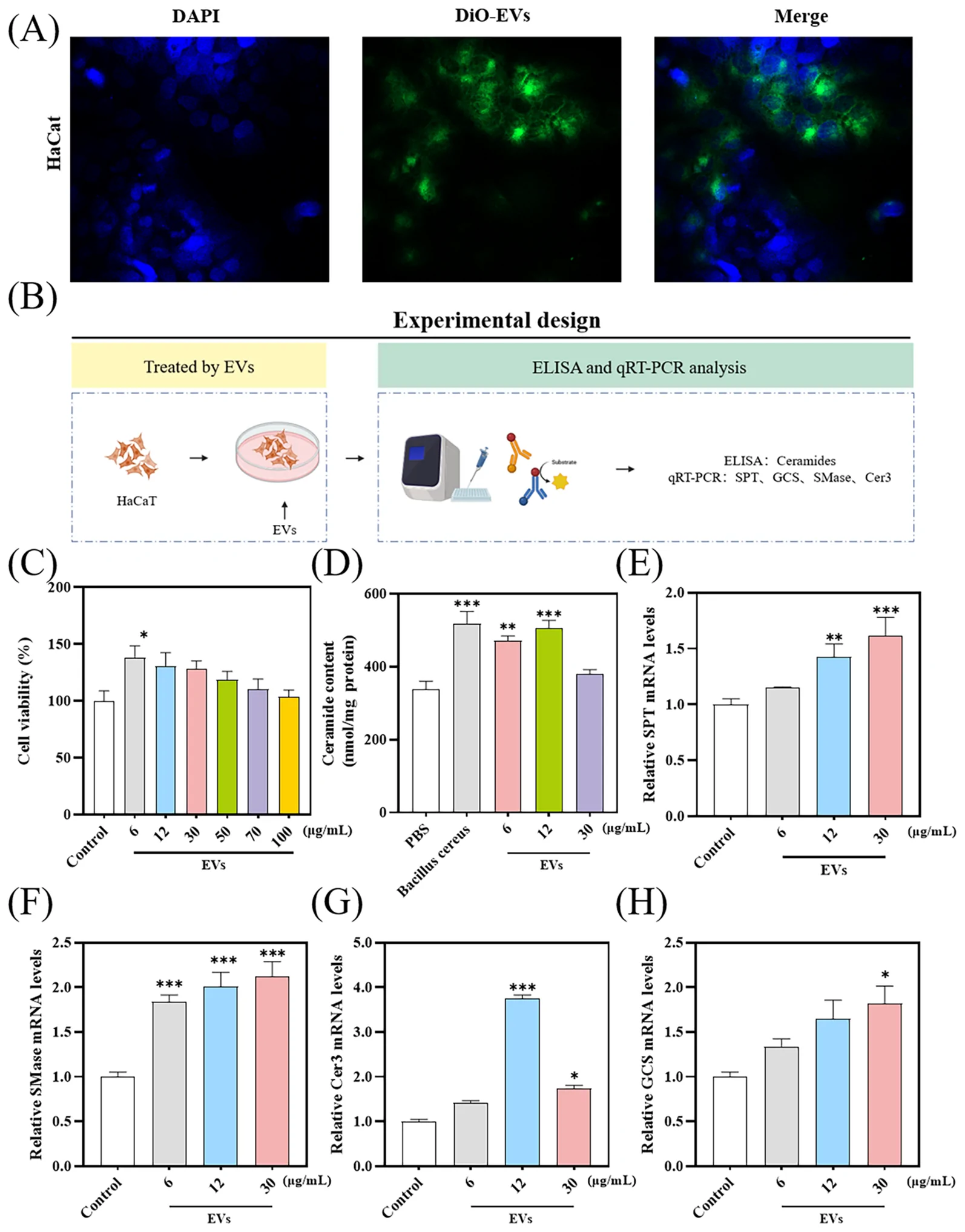
The cellular internalization, biocompatibility and the regulatory effect of EVs on the ceramide synthesis in HaCaT cells. **(A)** The confocal laser scanning microscopy images of cellular uptake in HaCaT cells treated with EVs. **(B)** The experimental design to evaluate the regulatory effect of EVs on the ceramide production and ceramide metabolism-related gene expression. **(C)** The cell viability of HaCaT cells treated with EVs. **(D)** The content of ceramides in HaCaT cells treated with EVs. **(E)** The mRNA expression levels of serine palmitoyltransferase (SPT) in HaCaT cells treated with EVs. **(F)** The mRNA expression levels of sphingomyelinase (SMase) in HaCaT cells treated with EVs. **(G)** The mRNA expression levels of ceramide synthase 3 (CerS3) in HaCaT cells treated with EVs. **(H)** The mRNA expression levels of glucocerebrosidase (GCS) in HaCaT cells treated with EVs. *p < 0.05, **p < 0.01, and ***p < 0.001.

Given their nanoscale dimensions, EVs may be able to enter target cells efficiently through endocytosis or membrane fusion, thereby enabling intracellular delivery of bioactive molecules. The cellular internalization of the EVs was first assessed in order to clarify the basic action prior to evaluating their further biological effects. The observation by confocal laser scanning microscopy revealed that, the DiO-labeled EVs (DiO-EVs) exhibited distinct green fluorescence signals predominantly localized in the cytoplasmic region and perinuclear areas after 2 hours of coincubation with HaCaT cells (**Figure 2A**). These observations suggested that DiO-labeled EVs were associated with HaCaT cells and exhibited intracellular localization after incubation.

Subsequently, the effect of EVs on cellular metabolic activity was evaluated using the CCK-8 assay as a preliminary assessment of biocompatibility. As shown in **Figure 2C**, the EVs at the tested concentrations ranging from 6 to 100 μg/mL exhibited comparable or slightly higher metabolic activity than the control group, suggesting that EVs exhibited no apparent cytotoxicity within the tested concentration range. Moreover, the low concentration of 6 μg/mL of EVs significantly enhanced the cellular metabolic activity, indicating that EVs have the potential to promote metabolic activity, and the effect was more pronounced at the low concentration.

### 3.3 Effects of EVs on ceramide production and ceramide metabolism-related gene expression

As the first line of defense against external insults, the maintenance of the skin barrier function is crucial for overall health. Ceramides are considered as the main lipid component in the physical barrier of the skin, and their levels directly determine the integrity and function of the skin barrier (28). Previous studies have shown that ceramide synthesis in the skin is primarily accomplished through three pathways, including the *de novo* synthesis pathway, the SMase-catalyzed pathway, and the GCS-mediated salvage pathway (29, 30). Therefore, we investigated the effect of EVs on the ceramide production and examined the transcriptional changes of several genes associated with ceramide metabolism (**Figure 2B**).

Since *Bacillus cereus* fermentation supernatant has been shown to be rich in SMase and can significantly enhance ceramide synthesis (31, 32), it was used as the positive control, while PBS served as the negative control for ceramide quantification in HaCaT cells. As expected, the fermentation supernatant of *Bacillus cereus* significantly increased the production of ceramides in HaCaT cells (**Figure 2D**). Additionally, EVs at concentrations of 6 μg/mL, 12 μg/mL, and 30 μg/mL all displayed promoting effects on the intracellular ceramide levels. Notably, 12 μg/mL EVs exhibited comparable efficacy to the positive control of *Bacillus cereus* fermentation supernatant in enhancing ceramide production. Further qPCR analysis of ceramide metabolism-related genes revealed that EVs upregulated the mRNA expression of SPT, SMase, CerS3, and GCS. Among these genes, the mRNA expression levels of SPT, SMase, and GCS generally exhibited an increasing tendency with increasing EVs concentrations. Notably, the strongest increase in CerS3 mRNA expression was observed at 12 μg/mL EVs, suggesting that CerS3 transcription was responsive to EV stimulation. However, further studies are required to determine whether this transcriptional alteration contributes to increased enzyme activity. Thus, EVs may influence ceramide-associated metabolic processes by modulating the transcriptional expression profile of multiple ceramide metabolism-related genes.

### 3.4 Effects of EVs on barrier integrity and barrier-associated gene expression in AD-like cellular models

**Figure 3:**
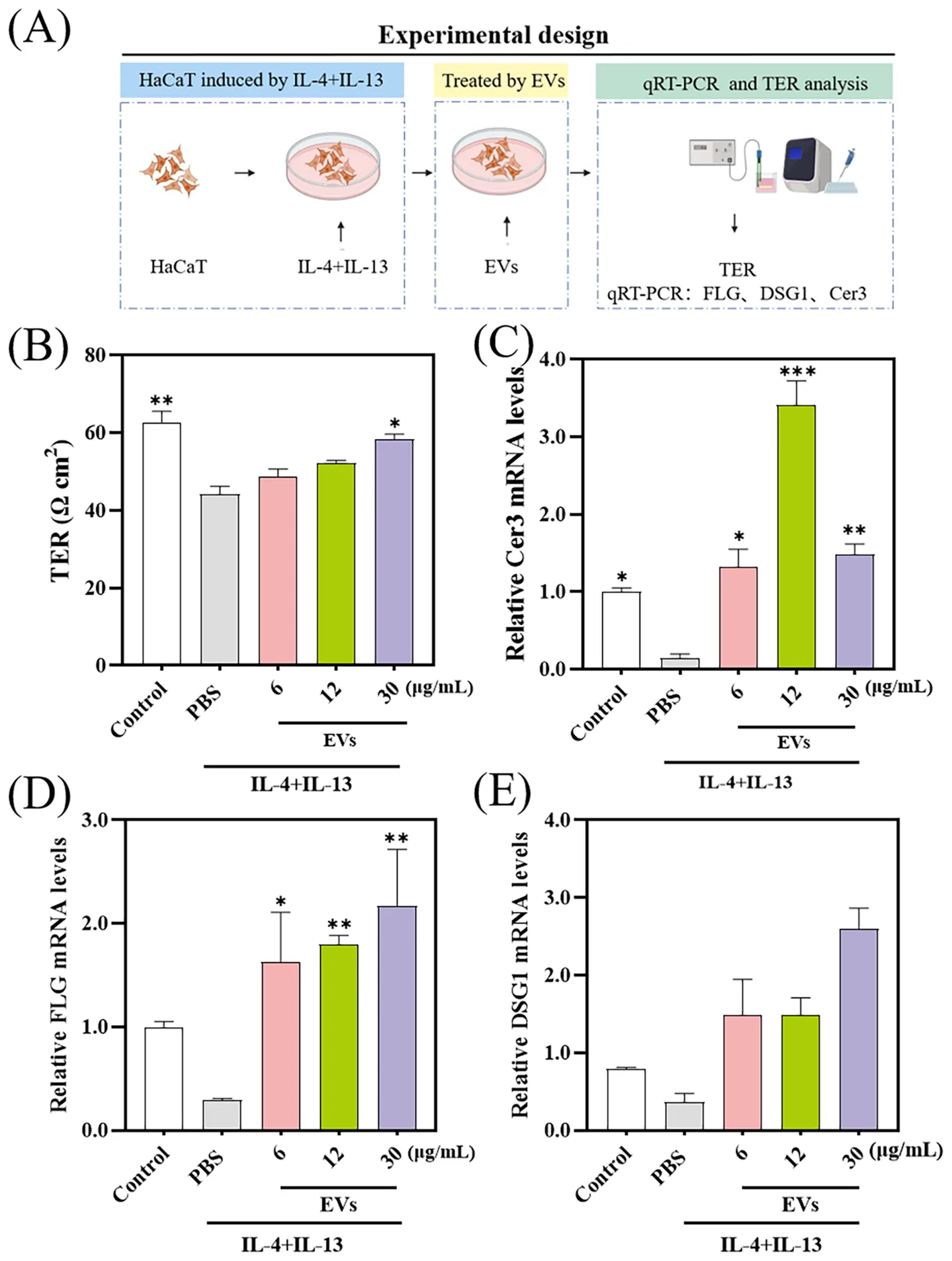
The reparative effects of EVs on barrier integrity and barrier-associated gene expression in AD-like cellular models. **(A)** The experimental design to evaluate the reparative effects of EVs on the barrier function. **(B)** The transepithelial electrical resistance (TER) analysis in AD-like cellular models after treatments of EVs. **(C)** The mRNA expression levels of ceramide synthase 3 (CerS3) in AD-like cellular models treated with EVs. **(D)** The mRNA expression levels of filaggrin (FLG) in AD-like cellular models treated with EVs. **(E)** The mRNA expression levels of desmoglein-1 (DSG1) in AD-like cellular models treated with EVs. *p < 0.05, **p < 0.01, and ***p < 0.001.

Previous studies have reported that skin barrier dysfunction serves as the critical initiating event in AD pathogenesis, leading to increased skin permeability, elevated transepidermal water loss, and heightened susceptibility to environmental irritants and allergens, which collectively trigger inflammatory responses (33, 34). The Th2-type immune response plays a central role in type 2 inflammation characteristic of AD, with its signature cytokines IL-4 and IL-13 recognized as key mediators of disease progression (35). These cytokines not only impair the barrier function of keratinocytes by reducing the expression of structural proteins, leading to increased skin permeability, but also activate eosinophils and mast cells, triggering an inflammatory response. Thus, we established an *in vitro* AD-like cellular model by treating HaCaT cells with 50 μg/mL IL-4 and 50 μg/mL IL-13, in order to systematically evaluate the potential roles of EVs in both enhancing barrier function and suppressing inflammation (**Figure 3A**).

As illustrated in **Figure 3B**, the transepithelial electrical resistance (TER) analysis was conducted in the AD-like cellular model. The significantly decreased TER values in the AD-like cellular model group, confirming barrier impairment and the successful construction of the model. The treatments with EVs at 6, 12, and 30 μg/mL concentrations induced dose-dependent recovery of TER values, demonstrating the capacity of EVs to restore epithelial barrier integrity. Subsequently, the quantitative PCR analysis of skin barrier-related genes provided molecular evidence that EVs effectively reversed suppression on transcriptional expression of several barrier-associated genes in the AD-like cellular model. It was clearly indicated that all the detected concentrations of EVs exhibited higher expression levels of CerS3 compared to control group and PBS group (**Figure 3C**). Remarkably, there was a dramatic 11.8-fold and 3.4-fold upregulation of CerS3 mRNA following treatment with 12 μg/mL EVs compared to control group and PBS group respectively, which closely paralleled the observed increasement in intracellular ceramide content previously. Moreover, treatment with EVs also elevated the expression levels of CerS3, FLG and DSG1 (**Figures 3D, E**). Overall, the increased expression of CerS3, FLG and DSG1 compared to both control and PBS group, showed that the restoration not only restored the impaired gene expression of these critical barrier proteins in the AD-like cellular model, but further enhanced their expression beyond normal physiological levels.

### 3.5 Effects of EVs on inflammatory gene expression in AD-like cellular models

**Figure 4:**
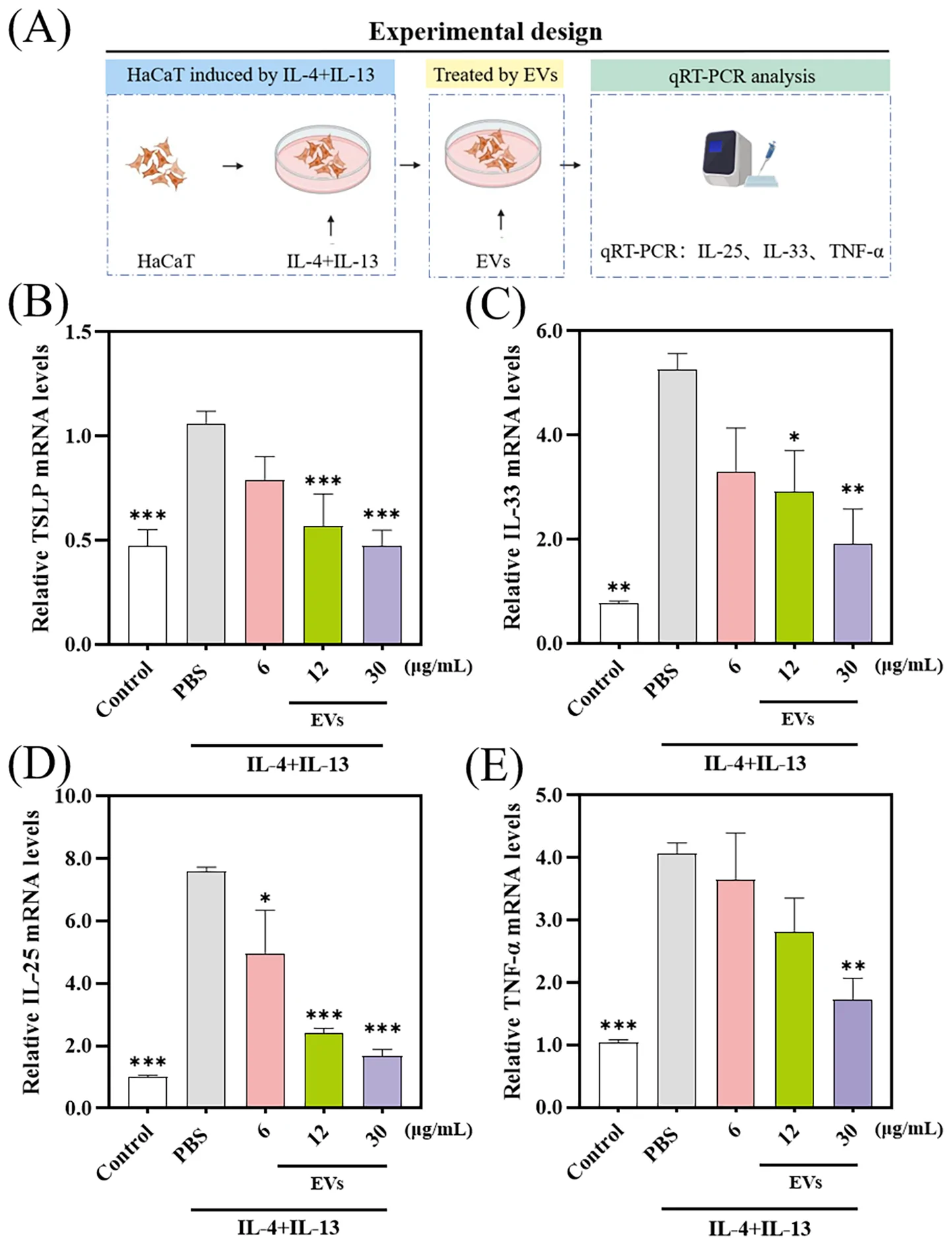
The inhibitory effect of EVs on inflammatory gene expression in AD-like cellular models. **(A)** The experimental design to evaluate the inhibitory effect of EVs on inflammatory gene expression. **(B)** The mRNA expression levels of thymic stromal lymphopoietin (TSLP) in AD-like cellular models treated with EVs. **(C)** The mRNA expression levels of interleukin-33 (IL-33) in AD-like cellular models treated with EVs. **(D)** The mRNA expression levels ofinterleukin-25 (IL-25) in AD-like cellular models treated with EVs. **(E)** The mRNA expression levels of tumor necrosis factor-α (TNF-α) in AD-like cellular models treated with EVs. *p < 0.05, **p < 0.01, and ***p < 0.001.

Studies have shown that IL-4 and IL-13 can significantly induce the production of various inflammatory factors in keratinocytes that play important roles in the pathogenesis of AD, including TSLP, IL-33, IL-25, and TNF-α (36). The AD-like cellular models induced by IL-4 and IL-13 were intervened by adding EVs to investigate the immunomodulatory effects of EVs (**Figure 4A**). The qPCR results revealed that IL-4 and IL-13 significantly stimulated key inflammatory mediators in the model group compared to untreated controls, as TSLP, IL-33, IL-25, and TNF-α were upregulated by 2.4 times, 5.1 times, 4.7 times and 3.6 times (p < 0.01)respectively (**Figures 4B–E**). The treatment with EVs produced a concentration-dependent inhibition of these inflammatory markers. The EVs at 30 μg/mL showed the strongest reduction in the mRNA expression levels, achieving 58.3% (p < 0.01), 62.3% (p < 0.01), 72.3% (p < 0.01) and 50.1% (p < 0.01) suppression of TSLP, IL-33, IL-25 and TNF-α respectively. Even at the lowest concentration of 6 μg/mL, EVs also significantly reduced IL-25 expression by 28.3% (p < 0.05). These findings collectively revealed that the EVs can simultaneously modulate the transcriptional expression of multiple inflammatory factors, suggesting a potential immunomodulatory role of EVs in AD-associated inflammation.

### 3.6 Alleviating effect of EVs on AD-like lesions in DNFB-induced mouse model

**Figure 5:**
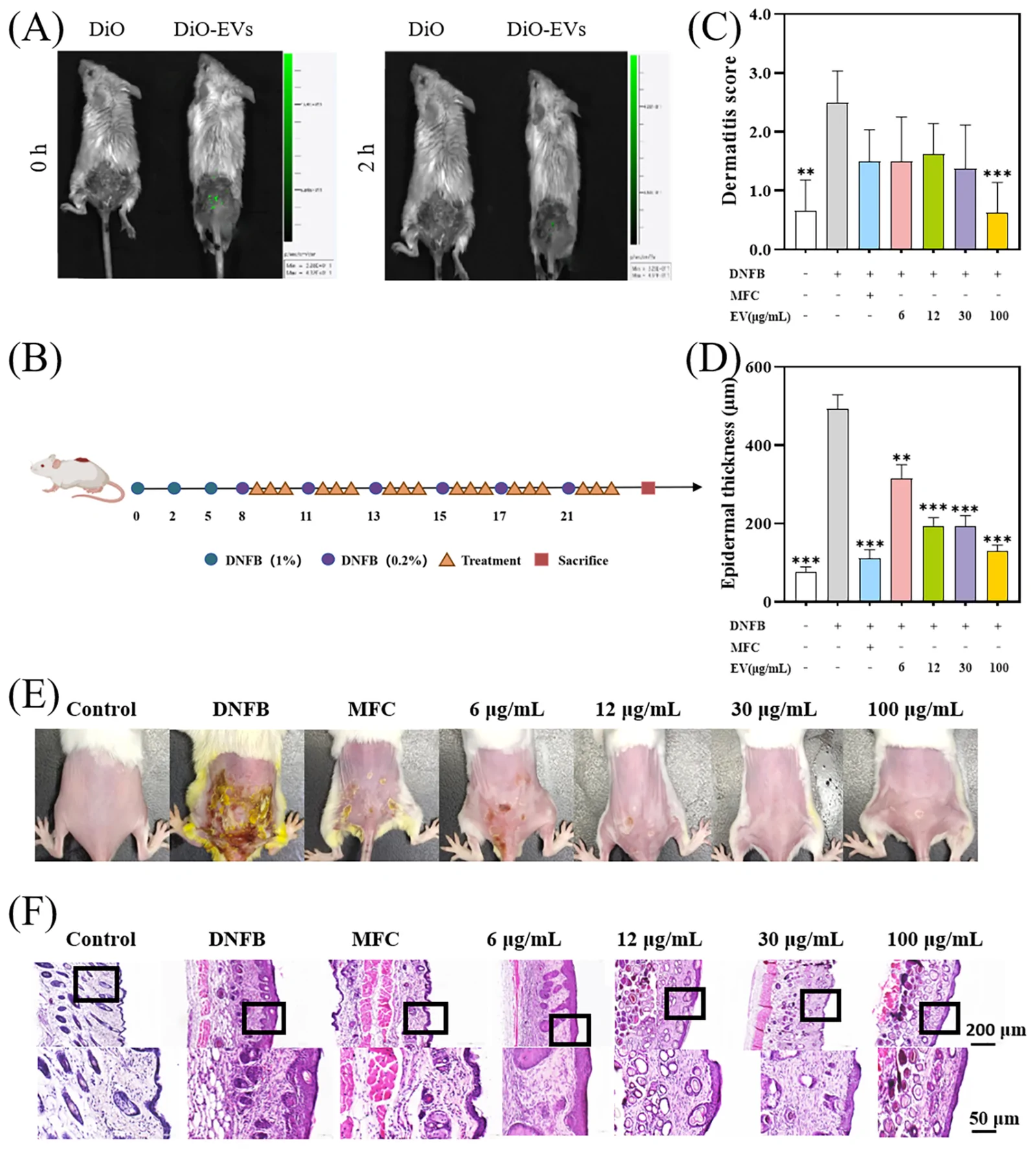
The alleviating effect of EVs on AD-like lesions in mice. **(A)** The transdermal capability of EVs. **(B)** The experimental design for the establishment and intervention of AD-like mouse model. **(C)** The clinical dermatitis score of AD mice. **(D)** The quantitative data on the epidermal thickness of AD mice. **(E)** The skin appearance images of AD mice. **(F)** The Hematoxylin and eosin (H&E) staining of AD mice. **p < 0.01, and ***p < 0.001.

To further evaluate the transdermal capability of EVs, the dorsal skin of BALB/c mice were depilated, followed by topical application of DiO-labeled EVs. *In vivo* fluorescence imaging was performed at 0 h and 2 h to examine the distribution of EVs in the skin tissue. The fluorescence signals indicated that fluorescently labeled EVs were retained and distributed at the application site after topical administration(**Figure 5A**).

In this study, the AD-like mice model was established using BALB/c mice subjected to repeated DNFB stimulation (**Figure 5B**). On the 7th day of modeling, the mice were regrouped by eliminating outliers to establish equivalent baseline conditions, with all groups showing no statistically significant differences in the clinical scores (p > 0.05), thus verifying consistent initial lesion severity. At the study endpoint of 24th day, the model group exhibited diffuse erythema, xerosis, scaling, crusting, and petechial hemorrhage (**Figure 5E**), with a clinical score of 2.4 ± 0.5 (**Figure 5C**), significantly higher than the control group, confirming successful establishment of the AD-like mice model. After 23 days of treatment, all groups administrated with EVs showed significant improvement in skin lesions. Notably, the EVs at 100 μg/mL showed superior efficacy with the clinical score reduced to 0.8 ± 0.3, which was better than the positive control of 0.1% mometasone furoate cream (MFC) group (1.5 ± 0.4), and the skin appearance closely resembled that of healthy controls. Histopathological analysis via H&E staining revealed marked epidermal hyperplasia in the model group, including acanthosis and hyperkeratosis (**Figure 5F**). The quantification results indicated that the model group exhibited an epidermal thickness of 520 ± 42 μm, approximately 7.2-fold greater than the control group (p < 0.001, **Figure 5D**). The intervention of EVs showed a dose-dependent alleviation of epidermal hyperplasia, with a thickness of 170 ± 25μm in the group of 30 μg/mL EVs and further reduced to 135 ± 20 μm in the group of 100 μg/mL EVs. Therefore, EVs can effectively repair the AD-like skin lesions induced by DNFB, and the therapeutic effect of the 100 μg/mL EVs group was comparable to that observed in the MFC group.

### 3.7 Restorative effects of EVs on the epidermal barrier of DNFB-induced AD mice

**Figure 6:**
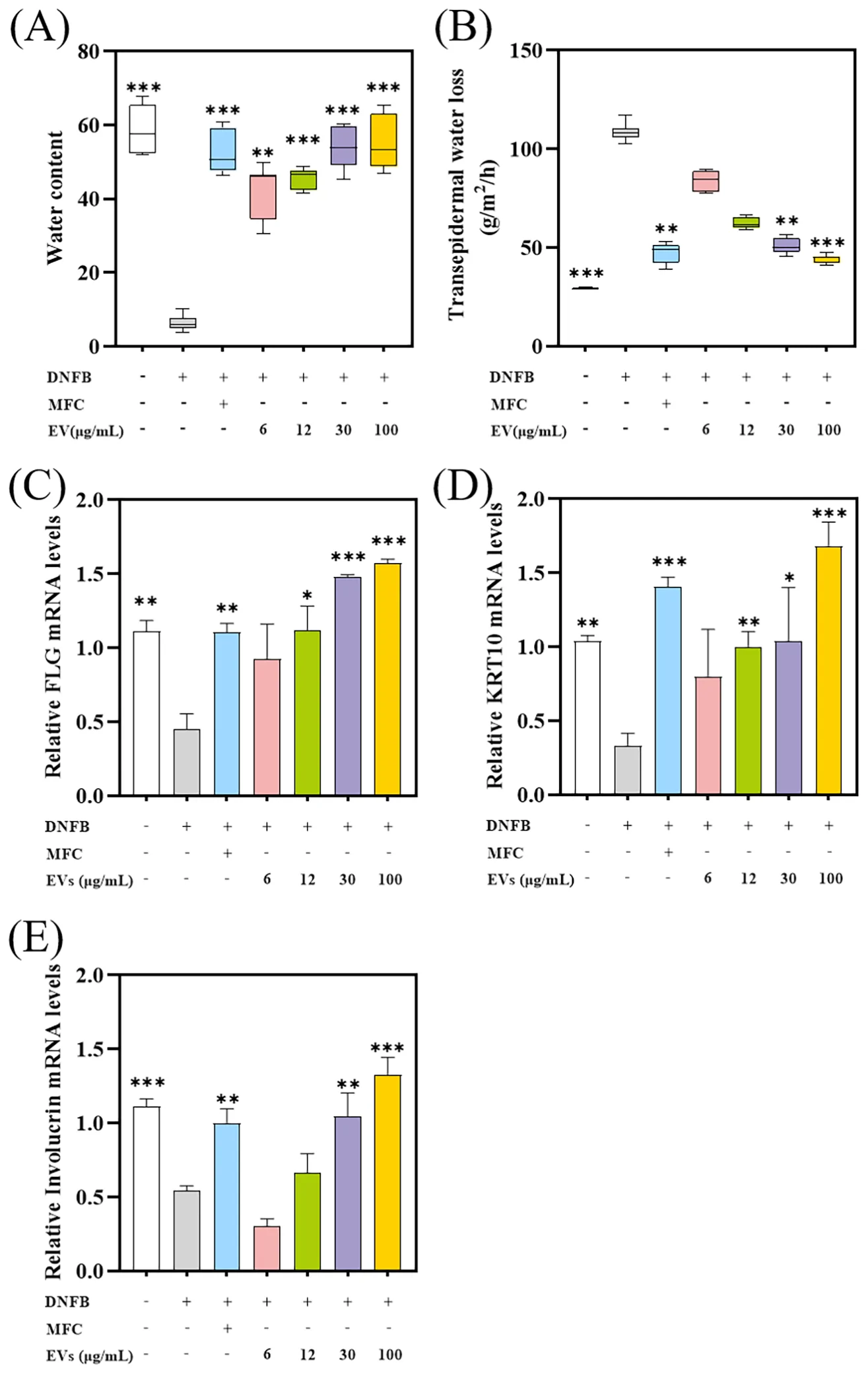
The restorative effects of EVs on the epidermal barrier of DNFB-induced AD mice. **(A)** The water contents of AD mice treated with EVs. **(B)** The transepidermal water loss (TEWL) of AD mice treated with EVs. **(C)** The mRNA expression levels of filaggrin (FLG) of AD mice treated with EVs. **(D)** The mRNA expression levels of keratin 10 (KRT10) of AD mice treated with EVs. **(E)** The mRNA expression levels of involucrin of AD mice treated with EVs. *p < 0.05, **p < 0.01, and ***p < 0.001.

The decrease in epidermal hydration and the increase in transepidermal water loss (TEWL) are the key pathological features of impaired skin barrier in AD. The skin barrier function was firstly evaluated by measuring the epidermal water content and TEWL in this study. As shown in **Figure 6A**, the epidermal water content in the model group induced by DNFB significantly decreased to 6.4 ± 1.9 AU (Arbitrary Unit), and the TEWL in the model group significantly increased to 108 ± 6 g/m²/h (p < 0.001, **Figure 6B**) from 29.2 ± 1.1 g/m²/h of control group, reflecting severe impairment of the barrier function. In the positive control group treated with MFC, the water content returned to 49 ± 4 AU, and TEWL decreased to 45 ± 3 g/m²/h, suggesting that glucocorticoids could partially restore the skin barrier integrity. Similarly, the EVs also showed synergistic improvement in hydration and barrier function in a concentration-dependent manner. Specially, the most significant therapeutic effects were observed with 100 μg/mL EVs treatment, which not only improved epidermal hydration to 55 ± 5 AU, but also achieved better TEWL reduction to 42 ± 2 g/m²/h, surpassing the effects of MFC.

Further qPCR analysis showed that the DNFB stimulation in the model group successfully induced the characteristic barrier gene downregulation of AD, including FLG, KRT10 and involucrin genes (**Figures 6C–E**). However, the EVs significantly reversed the gene inhibition, and the expression level of the high-dose group exceeded that of the control group, as the 100 μg/mL EVs increased the gene expression levels of FLG, KRT10 and involucrin to 1.36 times, 1.60 times, and 1.12 times that of the control group. Thus, the EVs can repair epidermal barrier of DNFB-induced AD mice through increasing epidermal hydration, decreasing transepidermal water loss, and promoting gene expression of skin barrier-related proteins.

### 3.8 Anti-inflammatory effect of EVs in the DNFB-induced AD mice

**Figure 7:**
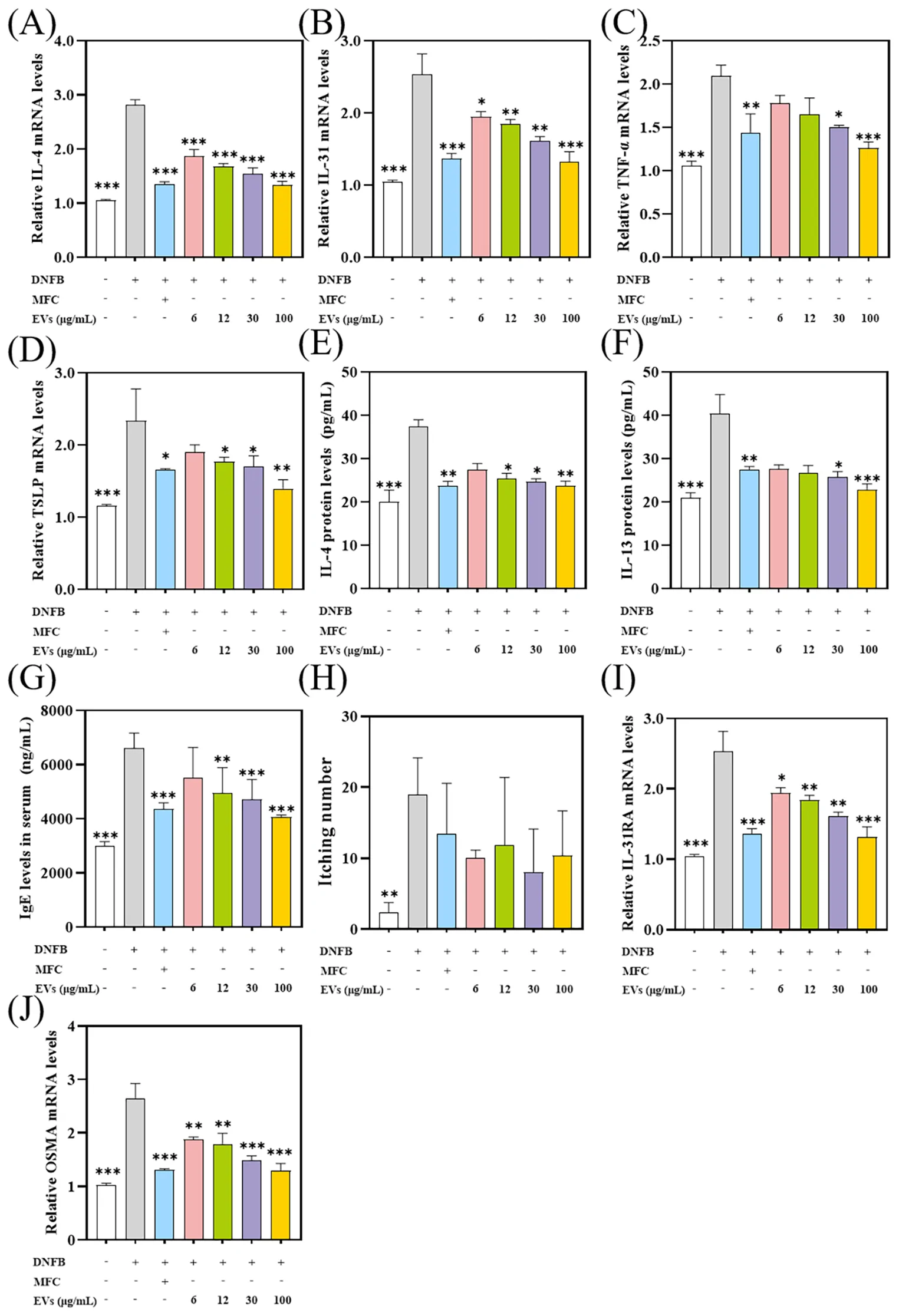
The anti-inflammatory and anti-pruritic effects of EVs in DNFB-induced AD mice. **(A–D)** The mRNA expression levels of interleukin 4 (IL-4), interleukin 31 (IL-31), tumor necrosis factor α (TNF-α) and thymic stromal lymphopoietin (TSLP) in skin tissues of AD mice treated with EVs. **(E, F)** The protein expression levels of IL-4 and interleukin 13 (IL-13) in skin tissues of AD mice treated with EVs. **(G)** The content of immunoglobulin E (lgE) in serum of AD mice treated with EVs. **(H)** The itching number of AD mice treated with EVs. **(I)** The mRNA expression levels of interleukin 31 receptor A (IL-31RA) in skin tissues of AD mice treated with EVs. **(J)** The mRNA expression levels of oncostatin M receptor (OSMR) in skin tissues of AD mice treated with EVs. *p < 0.05, **p < 0.01, and ***p < 0.001.

In this study, we detected the expression levels of key cytokines involved in the Th2-type immune response that the main inflammatory response of AD using qRT-PCR, to assess the anti-inflammatory effect of EVs in the AD mice. Compared to the control group, the expression levels of IL-4, IL-31, TNF-α, and TSLP in the model group significantly increased, while they were significantly decreased in the MFC group (**Figures 7A–D**). As for the treatments of EVs, the inhibitory effect on inflammatory factors became increasingly pronounced with escalating concentration of EVs, which was consistent with the observed improvement in dorsal skin lesions and reduced inflammatory cell infiltration in the AD mice. Particularly, the high concentration (100 μg/mL) EVs treatment group showed the most significant inhibitory effect on the inflammatory response, with no statistically significant difference compared to the MFC group (p > 0.05).

Then, parallel ELISA measurements of the protein contents of IL-4, IL-13, and IgE in the skin tissue and serum samples were conducted, which also confirmed the anti-inflammatory effect of EVs at the protein level. Similarly, compared to the control group, the protein expression levels of IL-4 and IL-13 in the mice skin tissues of the model group significantly increased, and the protein expression levels of these inflammatory factors significantly decreased after MFC treatment (**Figures 7E, F**). Additionally, EVs treatment significantly reduced the protein contents of IL-4 and IL-13, especially the inhibitory effect on inflammatory factors of EVs concentration at 30 μg/mL and 100 μg/mL was comparable to that of the MFC group. The total serum IgE in the model group was 2,480 ± 210 ng/mL, but it dropped to 1,050 ± 95 ng/mL after MFC intervention (**Figure 7G**). Likewise, EVs showed concentration-dependent inhibition on the IgE content of total serum, with 1,320 ± 120 ng/mL in the group of 30 μg/mL EVs and 920 ± 90 ng/mL in the group of 100 μg/mL EVs. These coordinated results at both transcriptional and protein levels demonstrate that EVs, particularly at high concentrations of 100 μg/mL, effectively suppressed Th2-type immune response and IgE production in the AD mice, achieving anti-inflammatory efficacy comparable to glucocorticoids of MFC.

### 3.9 Mitigating effect of EVs on the pruritic response in the AD-like dermatitis mice

Given that pruritus represents the core subjective symptom of AD and imposes significant burden on affected individuals, this study quantified itch intensity in the AD mice by counting the number of scratching actions within 30 minutes. EVs treatments showed a tendency to reduce scratching frequency in AD mice, although the differences among groups did not reach statistical significance. Studies have shown that IL-31 is a cytokine secreted by Th2-type immune cells, which binds to the heterodimeric receptor complex formed by interleukin 31 receptor A (IL-31RA) and oncostatin M receptor (OSMR), activating downstream signaling pathways such as JAK-STAT signaling pathways, thereby promoting the occurrence of inflammatory responses and itching (37, 38). Therefore, as the key components in the IL-31 signaling pathway, IL-31RA and OSMR, has been investigated in this study. qRT-PCR analysis clarified that the mRNA levels of IL-31RA and OSMR in the skin tissues of the model group were respectively 2.2 times and 2.8 times higher than those in the control group (**Figures 7I, J**), and the MFC treatment significantly downregulated the expression levels of these two genes. The gradient intervention of EVs also showed a concentration-dependent inhibition on the mRNA levels of IL-31RA and OSMR, with the 100 μg/mL EVs exhibiting the highest inhibitory effect. As recognized, compromised barrier integrity can directly lead to pruritus pathogenesis. Hence, the restoration of skin barrier integrity as demonstrated previously (**Figure 6**) may contribute to the observed alleviation of pruritus. Importantly, the marked downregulation of IL-31RA/OSMR mRNA expression highlighted that the EVs may also reduce the pruritus by modulating the IL-31 signaling pathway.

### 3.10 Exploration and mechanistic characterization of bioactive components in EVs

**Figure 8:**
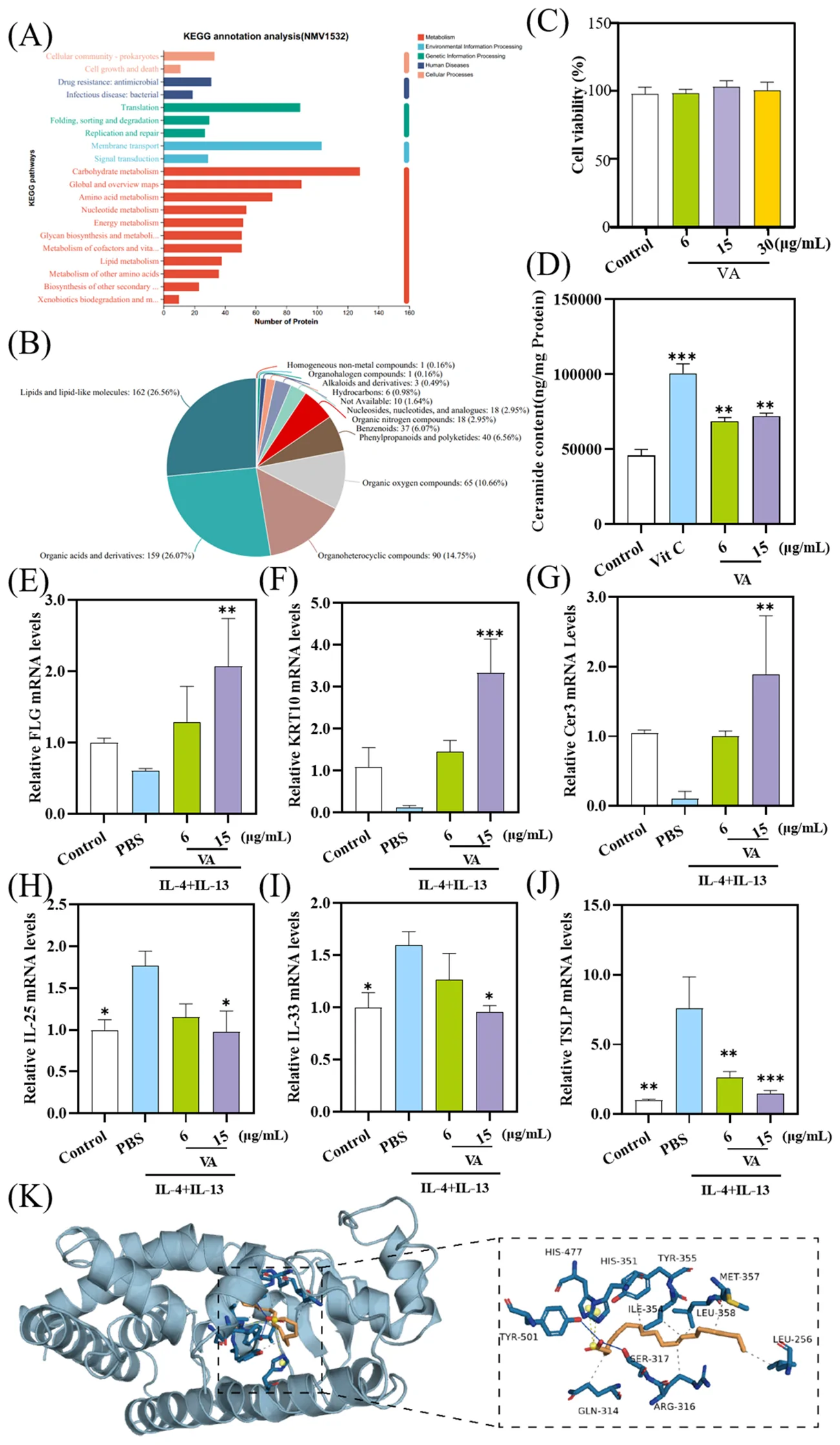
The exploration of bioactive components in EVs responsible for skin barrier enhancement and anti-inflammatory effect. **(A)** The proteomics analysis of EVs. **(B)** The metabolomics analysis of EVs. **(C)** The cell vitality of HaCat cells treated with 11-trans-octadecenoic acid (VA). **(D)** The content of ceramides in HaCat cells treated with VA. **(E–G)** The mRNA expression levels of filaggrin (FLG), keratin 10 (KRT 10), and ceramide synthase 3 (CerS3) respectively, in the AD-like cellular models treated with VA. **(H–J)** The mRNA expression levels of interleukin 4 (IL-25), interleukin 31 (IL-33), and thymic stromal lymphopoietin (TSLP) respectively, in the AD-like cellular models treated with VA. **(K)** The interaction between VA and peroxisome proliferator-activated receptor (PPAR) analyzed by molecular docking. *p < 0.05, **p < 0.01, and ***p < 0.001.

In order to explore the bioactive components from EVs, we conducted a proteomic analysis of EVs. The proteomic profiling identified a total of 1537 proteins, and functional annotation analysis revealed that proteins related to metabolism accounted for the highest proportion (**Figure 8A**). This finding prompted further metabolomic investigation, which demonstrated that lipids and lipids-like molecules represented the predominant metabolite class within EVs (**Figure 8B**). Through focused analysis of the lipid components, we found that C18 unsaturated carboxylic acid analogues were enriched in EVs. Based on this finding, we further selected 11-trans-octadecenoic acid (VA) as the most promising representative structural analogue of this lipid subclass for subsequent functional validation.VA is a recently characterized fatty acid derivative with established anti-inflammatory and barrier repair properties in both oral and intestinal mucosal environments (39–41). Considering the documented biological activities in other systems, we hypothesized that the VA might play a comparable role in cutaneous environments. Here, we investigated the anti-inflammatory and barrier repair functions of VA by a series of experiments *in vitro*.

We first obtained high-purity VA (≥99%) through *in vitro* chemical synthesis and evaluated its biological functions in HaCaT cells. The CCK-8 results showed that the cell viability of HaCaT cells treated with 6 and 15 μg/mL concentrations of VA did not show a significant decrease, suggesting excellent cellular compatibility (**Figure 8C**). The quantitative results of ceramides revealed that 6 μg/mL and 15 μg/mL VA increased cellular ceramide levels to 1.4 and 1.7 times that of the control group (p < 0.01), respectively, suggesting that VA significantly activated the ceramide synthesis pathway (**Figure 8D**). To further evaluate the potential of VA in barrier repair and anti-inflammatory therapy, we investigated its effects in the AD-like cellular models. The results manifested that VA intervention significantly upregulated the gene expression levels of protein factors closely related to skin barrier function (**Figures 8E–G**). Specifically, 6 μg/mL VA restored FLG, KRT10, and CerS3 to 1.3, 1.4, and 1.1 times that of the control group, respectively, while 15 μg/mL VA further upregulated them to 2.0 (p < 0.01), 3.1 (p < 0.001), and 1.9 times (p < 0.01). Further examination of the effect on inflammatory responses showed that VA treatment also significantly reduced the expression levels of inflammatory cytokines (**Figures 8H–J**). The inhibition rates for IL-25, IL-33, and TSLP of 6 μg/mL VA were 26.7%, 21.4%, and 59.6%, respectively, while 15 μg/mL VA increased the inhibition rates to 46.7%, 42.9%, and 74.5%, bringing the expression levels close to those of normal cells.

Molecular docking enables the prediction of ligand-protein interaction modes and binding characteristics based on geometric and energy matching principles (42). PPARs is a class of nuclear transcription factors activated by ligands, which are widely distributed in skin tissues and play an important role in regulating key cellular processes such as inflammation, cell proliferation, and differentiation, being implicated in various skin diseases (43, 44). PPARγ, as a subtype of PPARs, has been considered to be crucial in the skin for maintaining the permeability of the skin barrier, regulating cell proliferation and differentiation, and modulating inflammatory responses and antioxidant after ligand binding (45). Therefore, the activation of PPARγ is of great significance for the homeostasis of the skin, and the structural interaction between PPARγ and its ligands is essential for its biological function. Interestingly, the structure of VA closely resembles that of PPARγ agonists, suggesting that VA may exert its anti-inflammatory and skin barrier-restoring effects through PPARγ activation. Therefore, the molecular docking study was conducted to investigate the binding interactions between PPARγ and VA (**Figure 8K**). The molecular docking simulation generated nine distinct binding poses, among which the most energetically favorable conformation exhibited a binding affinity of -6.3 kcal/mol (**Supplementary Table 3**), indicating strong and stable binding between VA and PPARγ. Detailed analysis of the binding interactions revealed that VA formed crucial hydrogen bonds with two key residues: Y501 and S371. Furthermore, the binding interface showed extensive hydrophobic interactions involving multiple residues (L256, Q314, R316, Y335, L354, M357, and L358) of PPARγ, which significantly contributed to the stably combination of the PPARγ and VA. Additional stabilization was provided by the formation of salt bridges between VA and residues H351 and H477 of PPARγ. These computational results demonstrated that VA can effectively bind to PPARγ through a combination of specific molecular interactions, including hydrogen bonding, hydrophobic contacts, and electrostatic interactions. The observed binding mode shared characteristic features with known PPARγ ligands (46, 47), suggesting that VA may function as a potential PPARγ modulator. The comprehensive interaction profile provided valuable insights for understanding the molecular basis of the biological activity of EVs.

## 4 Discussion

AD has become a serious public health issue, characterized by its high incidence, prolonged course, and susceptibility to relapse (1, 3, 4). The complex pathogenesis of AD, coupled with the limitations of conventional pharmacotherapy, highlights the necessity for novel therapeutic strategies. Probiotics have been extensively applied for their beneficial health effects, such as enhancing barrier function and modulating the immune system (7, 8). As a representative probiotic species, *L. plantarum* has been reported to exert positive effects in treating human skin diseases (9). However, considering the potential risks associated with live bacteria, such as bacterial gene transfer or systemic infections in susceptible populations, strategies utilizing non-viable bacterial-derived postbiotics have been explored. Bacterial EVs represent a promising postbiotic agent that may offer benefits similar to live bacteria while minimizing associated risks. Therefore, based on the limitations of live bacterial therapy and the therapeutic potential of bacterial EVs, this study prepared EVs from *L. plantarum* to investigate their alleviating effects on AD. We found that the prepared EVs could be taken up by skin keratinocytes *in vitro*, promoting ceramide production and suppressing the expression of inflammatory factors. In an DNFB-induced AD-like mouse model, EVs achieved rapid transdermal absorption in the skin, alleviating AD symptoms and reducing itching by enhancing the epidermal barrier and modulating inflammatory responses. Strikingly, EVs treatment even exhibited significantly enhanced therapeutic effects over the positive control of mometasone furoate cream.

EVs possess unique physicochemical properties that may govern their biological activity (48). In our investigation, although the EVs in our study were similar to those in previous studies, presenting a regular spherical structure with a membrane, having a nanometer-sized particle size and carrying a negative charge, the specific values of their particle size (132.2 ± 5.8 nm) and charge (-11.54 ± 1.27 mV) were different from those reported in previous studies (49). Additionally, SDS-PAGE analysis revealed distinct protein banding patterns, suggesting strain-dependent compositional variations among bacterial EVs. These physicochemical differences likely stem from their bacterial origins, directly correlate with divergent biological functions. For instance, recent studies have shown that EVs derived from human skin commensals like *Staphylococcus aureus* (*S. aureus*) and *Cutibacterium acnes* played crucial roles in the pathogenesis of AD and acne vulgaris, respectively (50, 51). In contrast, our findings demonstrated that EVs prepared from *L. plantarum* were associated with amelioration of AD. The favorable nanoscale size of EVs can confer remarkable tissue penetration capabilities, making them particularly promising for dermatological applications. Our kinetic studies in HaCaT cells revealed rapid intracellular localization within just 2 hours, demonstrating their ability to be taken up by skin cells. Similarly, *in vivo* tracking in mice demonstrated that the fluorescently labeled EVs were retained and distributed at the application site after topical administration. This contrasted sharply with the pathological effects of *S. aureus-*derived EVs, which despite similar penetration profiles exacerbated inflammatory response (50). Importantly, the EVs obtained from *L. plantarum* not only achieved superior biodistribution but also exceptional safety characteristics. On the one hand, in contrast to viable bacterial preparations, *L. plantarum* EVs were inherently non-replicative, completely circumventing infection risks even following deep tissue penetration reaching subcutaneous compartments. Our cell viability test revealed that the EVs have excellent biocompatibility and can even promote cell metabolic activity at low concentrations. These findings provided compelling evidence for the safety profile of EVs as therapeutic carriers for AD intervention.

Given the intricate pathogenic mechanisms underlying AD, effective therapeutic intervention requires a multimodal strategy addressing key fundamental aspects, including epidermal barrier restoration, immune homeostasis modulation, and pruritus alleviation. Current understanding reveals that the pathogenesis of AD involves epidermal barrier impair, Th2-dominant immune dysregulation, and skin microbiome dysbiosis, and these components interact through complex mechanisms, creating a self-perpetuating cycle that drives disease progression (52). AD is frequently accompanied by epidermal barrier dysfunction, characterized by reduced expression of terminal differentiation proteins such as filaggrin and loricrin, along with impaired skin lipid membranes leading to increased transepidermal water loss (53). The observed decrease in epidermal thickness, improvement in skin water content, and reduction in TEWL in this study, indicated the beneficial effect of EVs on the epidermal barrier. Although the skin barrier is composed of physical, chemical, microbial, and immune barriers, the physical barrier serves as the outermost critical line of defense, primarily consisting of keratinocytes and intercellular lipids. Ceramides, the most distinctive and diverse components of stratum corneum lipids, play a pivotal role in maintaining skin barrier function (54). Our study found that EVs significantly increased ceramide levels both in HaCaT cells *in vitro* and in the skin of AD mice. Further investigation into the key enzymes involved in the three ceramide synthesis pathways, namely the *de novo* synthesis pathway, the SMase-catalyzed pathway, and the GCS-mediated salvage pathway, we observed the consistent upregulation of key enzymatic components, including SPT, SMase, CerS3, and GCS. These findings indicate that EV treatment increases ceramide levels and alters the transcriptional profile of genes involved in ceramide metabolism. While earlier work established that *L. plantarum* promoted ceramide biosynthesis via SMase secretion and SPT gene upregulation (55), we now showed that its derived EVs maintained this functional property. Additionally, the altered transcriptional expression of epidermal barrier-associated genes, including FLG, KRT10, and involucrin, further supported the potential involvement of EVs in regulating epidermal barrier function.

In addition to the dysfunction of the skin barrier, the pathogenesis of AD is also closely related to the imbalance of the immune system (56). Generally, the compromised epidermal barrier in AD allows external antigens to penetrate easily, stimulating keratinocytes to release alarmins including IL-25, IL-33 and TSLP (57). These alarmins activate dendritic cells and type 2 innate lymphoid cells (ILC2s), promoting Th2 cell polarization and subsequent production of cytokines such as IL-4 and IL-13, thereby establishing a Th2-dominant immune response. Such immune dysregulation in turn further disrupts skin barrier function, intensifies pruritus, and contributes to cutaneous microbiome disruption (58). In this study, we found that EVs treatment significantly reduced the transcriptional expression of inflammatory cytokines including IL-4, IL-25, IL-31, IL-33, TSLP and TNF-α, revealing that EVs may regulate AD-associated inflammatory responses. This immunomodulatory effect is consistent with previous findings *in vitro* by Kim et al., who demonstrated that EVs derived from *L. plantarum* possessed anti-inflammatory and immunoregulatory properties in human skin organ cultures, suggesting therapeutic potential for inflammatory skin disorders (59). Microbiome studies in AD revealed significant enrichment of *Staphylococcus aureus* alongside marked depletion of commensal bacteria with barrier-protective and immunomodulatory functions, such as *Staphylococcus epidermidis* and various *Lactobacillus* species e (4, 60). This dysbiosis can not only compromise the colonization resistance against pathogens but also upregulate Th2 cytokines through activation of pattern recognition receptors (PRRs). Furthermore, mediators produced by *S. aureus* such as enterotoxin B, can promote IL-31 expression (61), the key cytokine of pruritus in AD. Gene expression profiling of key IL-31 signaling mediators (IL-31RA and OSMR) showed significant downregulation following EVs intervention. Corroborating these molecular findings, EVs administration substantially decreased scratching frequency in murine models, providing functional evidence for its anti-pruritic efficacy in AD. Therefore, EVs represent a multifaceted treatment strategy for AD, simultaneously addressing key pathological features including barrier dysfunction, immune dysregulation, and pruritus. Nevertheless, the limitation of the present study should be acknowledged. Although EVs treatment exhibited beneficial effects on AD-associated phenotypes through improving epidermal barrier integrity, modulating ceramide metabolism-related responses, regulating inflammatory processes, and potentially alleviating pruritic manifestations, the current mechanistic investigations were primarily based on transcriptional analyses of related genes. Therefore, future studies involving protein-level validation, enzyme activity assays, and pathway-specific analyses are warranted to further elucidate the molecular mechanisms underlying EV-mediated regulation of barrier function, inflammation, and pruritus in AD. Considering the established biogenesis mechanisms of EVs, most of the bioactive molecules can be efficiently encapsulated by *L. plantarum* into EVs, and then directedly delivered to the host cells via various pathways (62). Through integrated proteomic and lipidomic analyses, we found that these EVs served as concentrated carriers of bacterial metabolites, with a striking enrichment of lipids and lipids-like molecules. Current understanding suggested that many of the lipids in EVs can not only modulate membrane curvature and fluidity and the capability of internalization into host cells, but also serve as key mediators of intercellular communication both between bacteria and host cells and among bacterial populations via protein recruitment and signal transduction (49). Most notably, the abundant presence of 11-trans-octadecenoic acid in EVs showed significantly promotion on the production of ceramides and the gene expression of epidermal barrier-related proteins FLG, KRT10, and CerS3, indicating a beneficial role in barrier repair. Additionally, 11-trans-octadecenoic acid markedly suppressed the expression of inflammatory cytokines IL-25, IL-33, and TSLP. Recent studies have revealed that specific octadecenoic acid derivatives exhibited anti-inflammatory and barrier-repair potential in different physiological sites. For example, 10-oxo-11-trans-octadecenoic acid showed significant anti-inflammatory effects in the oral environment (39). 10-oxo-6-cis, trans-octadecadienoic acid exhibited notable anti-inflammatory efficacy in the gut environment (40), while 10-hydroxy-cis-12-octadecenoic acid has been confirmed to enhanced intestinal epithelial barrier function (41). Consistent with previous findings in intestinal and oral environments, our results further demonstrated that 11-trans-octadecenoic acid, as one of octadecenoic acid derivatives, can also promote barrier restoration and inhibit inflammatory responses in the skin environment. Furthermore, since 11-trans-octadecenoic acid and PPARγ agonists shared highly similar structures, the molecular docking between 11-trans-octadecenoic acid and PPARγ was encouraged to validate the of action mode of 11-trans-octadecenoic acid. Surprisingly, there existed an effective binding relationship between 11-trans-octadecenoic acid and PPARγ. This binding was achieved through a series of unique molecular interactions, including hydrogen bonds, hydrophobic forces, and electrostatic interactions. These observations implied that the alleviating effects of EVs on AD, mediated through epidermal barrier repair and inflammation suppression, could be related to their intrinsic content of 11-trans-octadecenoic acid, which functions as a PPARγ agonist capable of activating the PPARγ signaling pathway. The mechanistic basis for these effects is supported by extensive evidence from prior research on the role of PPARγ in skin homeostasis and immunoregulation (45). As reported, PPARγ activation can initiate a transcriptional program that drive keratinocytes toward terminal differentiation (63). This process is characterized by a shift from proliferation to differentiation, leading to the robust expression of key structural proteins essential for barrier formation, including keratins, filaggrin, involucrin, loricrin. Concurrently, terminally differentiating keratinocytes can synthesize and secrete ceramides for maintaining both water retention and permeability barrier function (64). The integrated outcome is a reinforcement of the stratum corneum structure, in which cornified envelopes composed of the expressed proteins are embedded within a lamellar lipid matrix. Therefore, the promotion of ceramides and the increase in the gene expression of barrier-related proteins (FLG, KRT10, and CerS3) in this study confirmed the skin barrier repair effect produced by 11-trans-octadecenoic acid through the activation of PPARγ. Furthermore, 11-trans-octadecenoic acid exerted anti-inflammatory effects by suppressing alarmins such as IL-25, IL-33, and TSLP in keratinocytes, which are initiators of type 2 immunity in skin. This is consistent with reports that PPARγ activation inhibited NF-κB signaling and pro-inflammatory cytokine release (65). Specifically, the mice treated with PPARγ agonists revealed reduced expression of cytokines IL-4, IL-5, and IL-13, while mice without PPARγ agonists exhibited enhanced release of epithelial-derived alarm proteins including IL-25, IL-33, and TSLP as well as NF-kB activation. Moreover, PPARγ activation can also polarize macrophages toward the M2 phenotype, dampen dendritic cell maturation and T cell priming capacity, and promote regulatory T cell (Treg) differentiation, collectively contributing to resolution of skin inflammation (45, 65). Overall, our study identified 11-trans-octadecenoic acid as a critical effector molecule underlying the beneficial crosstalk between *L. plantarum* EVs and host skin homeostasis. Its activity is likely mediated through activation of PPARγ signaling, leading to enhanced barrier repair and suppression of inflammatory responses. These results stressed the potential of bacterial EVs as a novel therapeutic strategy for the treatment of AD and other related inflammatory skin disorders. Furthermore, although VA was identified as a candidate bioactive component within EVs, its direct contribution to EVs-mediated therapeutic effects remain to be further validated.

## 5 Conclusion

In summary, we developed the EVs derived from *L. plantarum* for AD alleviation. Our findings demonstrated that these EVs exhibited excellent cellular uptake and skin penetration capabilities. Remarkably, the EVs effectively promoted ceramide production, enhanced the epidermal barrier, modulated immune responses, and alleviated pruritus, collectively contributing to significant AD mitigation. Furthermore, our study suggested these therapeutic effects may be associated with their intrinsic lipid-based bioactive components. These findings provided novel insights into probiotic-based AD therapeutics, highlighting the therapeutic potential of EVs in dermatological disease management.

## Data Availability

The datasets presented in this study can be found in online repositories. The names of the repository/repositories and accession number(s) can be found in the article/**Supplementary Material**.
