# Supplementary material for: Extracellular vesicles from Lactobacillus plantarum alleviate DNFB-induced atopic dermatitis in mice by reconstructing the skin barrier and inhibiting inflammation
Source: Front Immunol. 2026 Aug 11;17:1873193. doi: 10.3389/fimmu.2026.1873193 (PMC13503329; doi:10.3389/fimmu.2026.1873193)
Supplement: Supplementary file 1 [file Supplementaryfile1.docx]

Supplementary Material

# 1 Supplementary Figures and Tables

**Supplementary Table S1.** The primer sequences used in quantitative polymerase chain reaction (qPCR) analysis of gene expression in HaCat cells.

| Primer name | Sequence（5’-3’） |
| --- | --- |
| SPT-F | TGGAAGAGAGCACTGGGTCT |
| SPT-R | GCTACCTCCTTGATGGTGGA |
| CerS3-F | CCAGGCTGAAGAAATTCCAG |
| CerS3-R | AACGCAATTCCAGCAACAGT |
| SMase-F | CGTCAGCATCCTCACGTACA |
| SMase-R | TGGCTCTTGGTCTTGGCTTT |
| GCS-F | ATGTGTCATTGCCTGGCATG |
| GCS-R | CCAGGCGACTGCATAATCAAG |
| CerS3-F | CCAGGCTGAAGAAATTCCAG |
| CerS3-R | AACGCAATTCCAGCAACAGT |
| GAPDH-F | GGAAGCTTGTCATCAATGGAAATC |
| GAPDH-R | TGATGACCCTTTTGGCTCCC |
| KRT10-F | CCTGGCTTCCTACTTGGACA |
| KRT10-R | TTGCCATGCTTTTCATACCA |
| FLG-F | TGAAGCCTATGACACCACTGA |
| FLG-R | TCCCCTACGCTTTCTTGTCCT |
| DSG1-F | TCCCATTTTTGATGATCTGTTGTG |
| DSG1-R | GCTCAAAGCCAGCTGCACTA |
| TSLP-F | TTCCTGTGGACTGGCAATGAG |
| TSLP-F | TTCCTGTGGACTGGCAATGAG |
| IL-33-F | TGTCACATTGGGCAAAGTT |
| IL-33-R | CAGTAAGCAGTGTTATCAGGAA |
| TNF-α-F | CCTCTCTCTAATCAGCCCTCTG |
| TNF-α-R | GAGGACCTGGGAGTAGATGAG |
| GAPDH-F | GGAAGCTTGTCATCAATGGAAATC |
| GAPDH-R | TGATGACCCTTTTGGCTCCC |

**Supplementary Table S2**. The primer sequences use in quantitative polymerase chain reaction (qPCR) analysis of gene expression in atopic dermatitis mice.

| Primer name | Sequence（5’-3’） |
| --- | --- |
| IL-4-F | GGTCTCAACCCCCAGCTAGT |
| IL-4-F | GCCGATGATCTCTCTCAAGTGAT |
| IL-31-F | TCGCTCAACACTTTGACTTTCT |
| IL-31-R | TCAGCAGACGAATCAATACAGC |
| TNF-α-F | CTGAACTTCGGGGTGATCGG |
| TNF-α-R | GGCTTGTCACTCGAGTTTTGAGA |
| TSLP-F | ACGGATGGGGCTAACTTACAA |
| TSLP-R | AGTCCTCGATTTGCTCGAACT |
| FLG-F | ATGTCCGCTCTCCTGGAAAG |
| FLG-F | TGGATTCTTCAAGACTGCCTGTA |
| KRT10-F | GTGTCCACTGGTGATGTGAATGTGG |
| KRT10-R | TTCTGTTTCTGCCAAGGAGGCTTCC |
| Cer3-F | CTGGCTTCCTCCAACAATAAAGTGG |
| Cer3-R | TCAAGTTACACTTCTTTGCCAGTCC |
| IL-31RA-F | TCGCTCAACACTTTGACTTTCT |
| IL-31RA-F | TCAGCAGACGAATCAATACAGC |
| OSMA-F | GATTCGCATCACAGCCAACAA |
| OSMA-R | CCAGATACGGGCTCCCAAGA |
| GAPDH-F | CATCACTGCCACCCAGAAGACTG |
| GAPDH-R | ATGCCAGTGAGCTTCCCGTTCAG |

**Supplementary Table S3**. The binding affinity parameters of the nine distinct binding poses generated by molecular docking simulation.

| **Mode** | **Affinity (kcal/mol)** | **RMSD l.b.** | **RMSD u.b.** |
| --- | --- | --- | --- |
| 1 | -6.3 | 0.000 | 0.000 |
| 2 | -6.2 | 1.596 | 3.584 |
| 3 | -6.2 | 1.465 | 3.167 |
| 4 | -6.2 | 5.595 | 10.238 |
| 5 | -6.2 | 4.485 | 10.984 |
| 6 | -6.1 | 0.856 | 1.672 |
| 7 | -6.1 | 4.107 | 6.422 |
| 8 | -6.1 | 4.849 | 10.832 |
| 9 | -6.0 | 4.432 | 10.305 |

**Supplementary Figure 1.** The PPAR structure obtained through homology modeling.

**Supplementary Figure 2.** Molecular weight analysis of Lactobacillus plantarum extracellular vesicles (EVs) by SDS-PAGE. Lanes 1 to 3 on the left are parallel samples of L. plantarum EVs, and lane 4 is the protein marker.

**2 Data availability**

All relevant research data are available upon reasonable request. All raw proteomic data have been deposited in the iProX database (accession number: PXD081555), and metabolomic datasets have been submitted to the MetaboLights database (accession number: MTBLS15122).
